# Morpho-Functional Characterisation of the Rat Ventral Caudal Nerve in a Model of Axonal Peripheral Neuropathy

**DOI:** 10.3390/ijms24021687

**Published:** 2023-01-14

**Authors:** Eleonora Pozzi, Laura Monza, Elisa Ballarini, Mario Bossi, Virginia Rodriguez-Menendez, Annalisa Canta, Alessia Chiorazzi, Valentina Alda Carozzi, Luca Crippa, Paola Marmiroli, Guido Cavaletti, Paola Alberti

**Affiliations:** 1 Experimental Neurology Unit, School of Medicine and Surgery, University of Milano-Bicocca, 20900 Monza, Italy; 2 NeuroMI (Milan Center for Neuroscience), 20126 Milan, Italy

**Keywords:** caudal nerve, anatomy, morphology, light microscopy, electron microscopy, nerve conduction studies, neuropathy, neurotoxicity, chemotherapy induced peripheral neuropathy, chemotherapy induced peripheral neurotoxicity

## Abstract

Peripheral Neuropathies (PN) are common conditions whose treatment is still lacking in most cases. Animal models are crucial, but experimental procedures should be refined in some cases. We performed a detailed characterization of the ventral caudal nerve to contribute to a more effective assessment of axonal damage in future PN studies. PN was induced via weekly systemic injection of a neurotoxic drug (paclitaxel); we compared the control and PN-affected rats, performing serial neurophysiological evaluations of the caudal nerve for its entire length. On the same nerve portions, we performed light microscopy and ultrastructural pathological observations to assess the severity of damage and verify the integrity of the surrounding structures. Neurophysiological and morphological analyses confirmed that a severe axonopathy had ensued in the PN group, with a length-dependent modality, matching morphological observations. The site of neurophysiological recording (e.g., distance from the base of the tail) was critical for achieving useful data. A flexible experimental paradigm should be considered in animal studies investigating axonal PN, particularly if the expected severity is relevant; the mid-portion of the tail might be the most appropriate site: there damage might be remarkable but neither as extreme as at the tip of the tail nor as mild as at the base of the tail.

## 1. Introduction

Peripheral neuropathies (PN) are common conditions affecting 2–3% of the world population, and they can seriously interfere with patients’ daily activities and quality of life [1]. PN have many different causes [2] and an efficacious curative, not only symptomatic, treatment still lacks for many of them [3]. Therefore, a multidisciplinary effort is required to investigate novel potential treatments, relying on a strong biological rationale: a bench-to-bedside approach (and vice versa) is crucial. The majority of PN (e.g., metabolic, genetic, toxic) leads to length-dependent distal axonal damage [4]; when going back to the bench side to study a length-dependent axonopathy, the caudal nerve is an ideal anatomical site to be tested in PN rat models since it is very distal and more than 50% longer than the sciatic nerve, allowing to better grasp length-dependency [5].

The reason to rely on preclinical models, in fact, is that preclinical in vivo research allows morphological and biomolecular analyses on peripheral nerves that cannot be easily performed in the clinical setting [6,7]. To maintain a high translational profile (i.e., to have a “decoy” that enables the transferal of morphological and biomolecular data from the bench to the bedside), nerve conduction studies (NCS) are the ideal investigational tool to translate preclinical results into the human condition; NCS, in fact, can be similarly performed at the bench and bedside [8,9]. We recently described a ready-to-use protocol to perform NCS in rat models of PN [10], relying on the evaluation of the digital and caudal nerves. In our previous work we evidenced that, in the case of severe length-dependent processes, the most distal portion of the caudal nerves can be so severely damaged that traces are not recordable, thus undermining the usefulness of recordings themselves at this level [10]. A possible way to overcome this methodological limitation would be to test the nerve at different sites so that the optimal recording option can be selected.

This study aims to provide an extensive morpho-functional characterization of the ventral caudal nerve in healthy and PN-affected rats (the control [CTRL] and PN group, respectively), relying on a robust model we already published: relevant axonal damage is induced via chronic administration of a neurotoxic agent (paclitaxel, PTX) [11]. We selected this model of length-dependent neuropathy to test our methodological approach to axonal damage ranging from mild to severe in the same experiment.

## 2. Results

### 2.1. NCS

**Figure 1:**
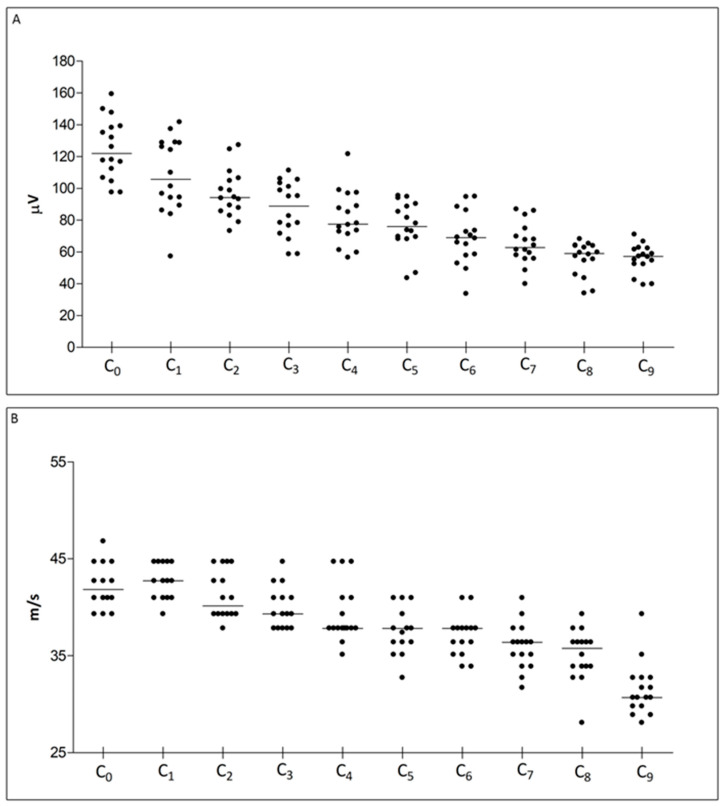
Distribution of NCS parameters at baseline, showing values at each site for the whole studies population. In (**A**) sensory nerve action potential (SNAP) amplitude data is shown, and in (**B**) sensory conduction velocity (SCV) data. Recordings were obtained following the montage described in methods section (C_0_ was obtained at the base of the tail and then the montage was placed distally in the adjacent segment C_1_, and subsequently recordings were repeated for the whole length of the tail up to the tip).

At baseline, the caudal nerves were assessed for the whole length of the tail. Data obtained from all animals are shown in Table 1 and represented in Figure 1; in order to explore the normal variation of parameters along the length of the tail at baseline, we first considered the whole population as one group (e.g., data from both groups are pooled together). Nerve segments were named going from proximal to distal (e.g., C_0_ equals the base of the tail, C_1_ equals 1 cm from the base of the tail, C_2_ equals 2 cm from the base of the tail, etc.).

**Figure 2:**
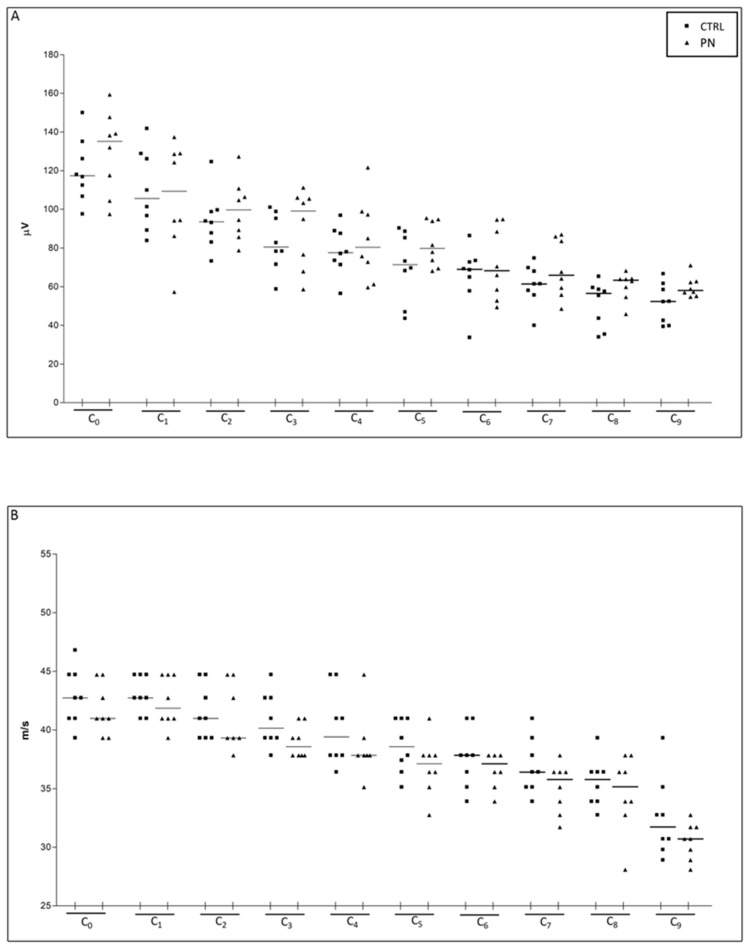
NCS parameters at baseline, ensuring homogeneity between the 2 groups. In (**A**) sensory nerve action potential (SNAP) amplitude data is shown, and in (**B**) sensory conduction velocity (SCV) data. Recordings were obtained following the montage described in Figure 1 (C_0_ was obtained at the base of the tail, then the montage was placed distally in the adjacent segment C_1_, and subsequently recordings were repeated for the whole length of the tail up to the tip). CTRL: control group; PN: peripheral neuropathy group.

Both sensory nerve action potential (SNAP) amplitude and sensory conduction velocity (SCV) progressively diminish, following the progressive nerve branching (e.g., in the most distal portions, the total number of large myelinated fibers, detected via NCS, physiologically decreases). When comparing data from adjacent segments (e.g., C_0_ vs. C_1_, C_0_ vs. C_2_, …) a statistically significant difference in NCS parameters was observed when applying at least 1 cm of distance between two nearby recording montages, thus meaning that when performing serial recordings a 1 cm distance is required to grasp nerve portion with different fiber composition. We then divided the whole population into the two groups (CTRL and PN) before starting treatment, ascertaining that data obtained from homologous sites (i.e., C_0_ vs. C_0_, C_1_ vs. C_1_, …) showed no difference between the 2 groups, as shown in Figure 2.

**Figure 3:**
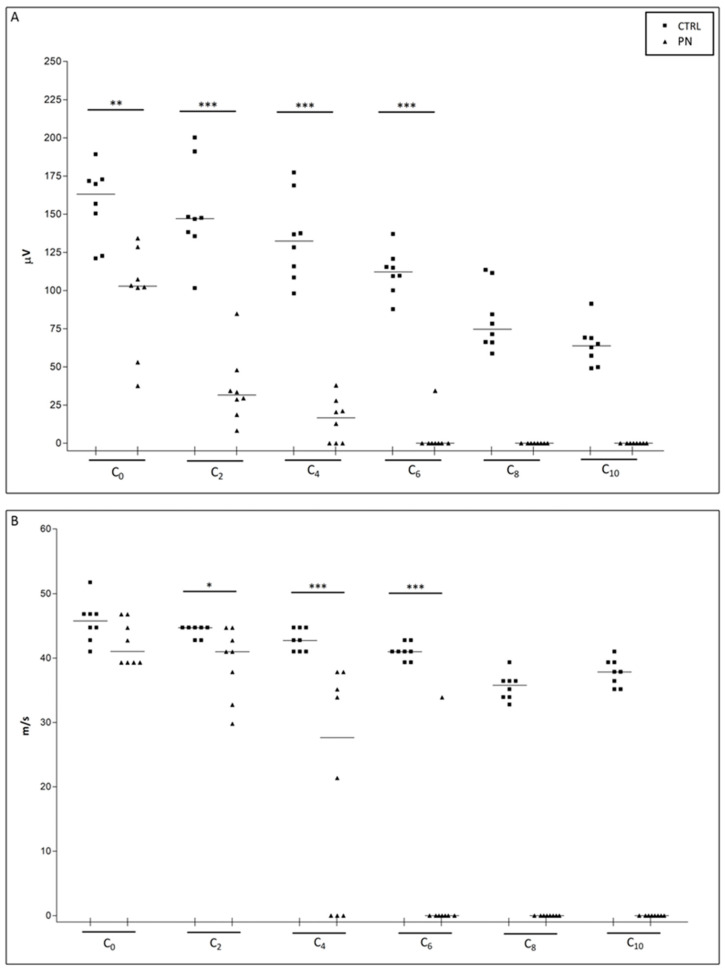
NCS at the end of treatment. In (**A**) sensory nerve action potential (SNAP) amplitude data is shown and in (**B**) sensory conduction velocity (SCV) data. Recordings were obtained following the montage described in Figure 1 (C_0_ was obtained at the base of the tail, then the montage was placed distally in the adjacent segment C_1_, and subsequently recordings were repeated for the whole length of the tail up to the tip). CTRL: control group; PN: peripheral neuropathy group. *: *p*-value < 0.05; **: *p*-value > 0.01; ***: *p*-value < 0.001.

At the end of treatment, NCS were repeated testing serially segments that were 1 cm apart from each other—for the reason stated above -, starting from the base of the tail (e.g., C_0_, then C_2_, then C_4_, …). Alterations compatible with axonal damage, represented by a significant SNAP amplitude decrease, were observed as soon as C_0_ recordings in PN animals; moreover, a length-dependent increase in severity was observed as we progressed with serial recordings going from proximal to distal. SCV was altered, in PN animals starting from the level of C_2_ segment and in the sites distal to it. Furthermore, some animals showed such severe damage starting from C_4_ segment that in some cases no SNAP was recorded (at C_4_ traces were not recordable in 3 animals out of 8). The damage severity progressively increased going distally up to the point that at the level of C_8_ no traces were recordable in all PN animals. Data and statistical analyses (Mann-Whitney U-test) are summarized in Figure 3.

### 2.2. Light Microscopy of the Whole Tails

**Figure 4:**
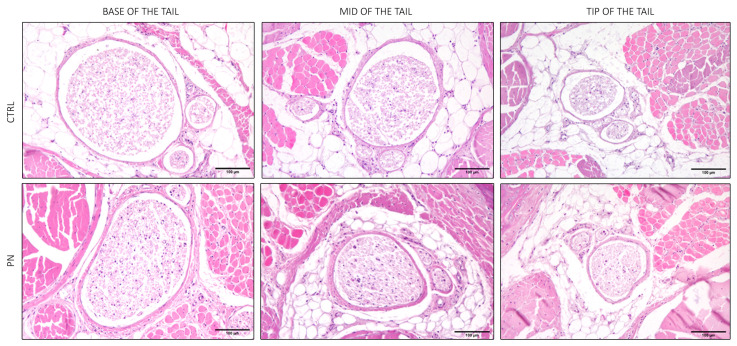
Light microscopy of significant sections of the whole tail (haematoxylin and eosin). In the upper panel, representative images from control (CTRL) animals, and in the bottom panel representative images of the peripheral neuropathy (PN) animals are shown. CTRL: control group; PN: peripheral neuropathy group. Scale bars represent 100 µm.

The main feature that can be highlighted by observing the anatomy of the tail in CTRL animals (Figure 4), is that the nerves, surrounded by the tail muscles, progressively decreased in diameter, as expected since nerves progressively branch to reach the target of interest. The PN group showed the same features, and a careful examination of the lateral tail veins did not evidence any local reaction to iv administrations, confirming that axonopathy in PN animals was not due to focal nerve damage, potentially induced by the repeated iv administration of the neurotoxic drug but rather to the drug systemic neurotoxic effect.

### 2.3. Light Microscopy of the Isolated Ventral Caudal Nerve

**Figure 5:**
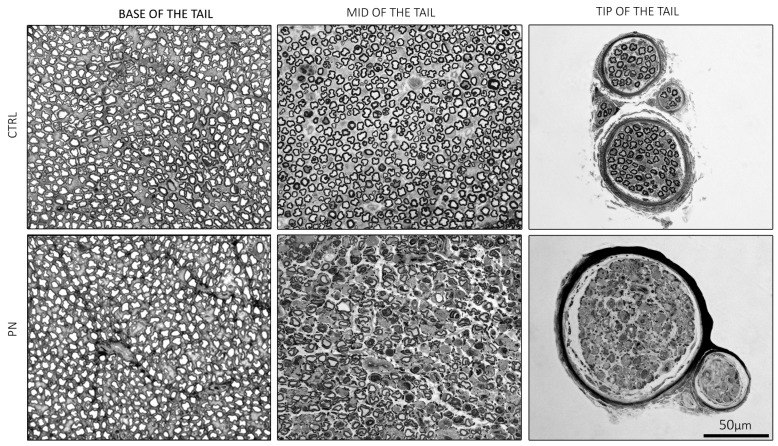
Representative images of ventral caudal nerves specimens (toluidine blue). In the upper panel, representative images of the control (CTRL) animals, and in the bottom panel representative images of the peripheral neuropathy (PN) animals are shown. Scale bar shown in the bottom right quadrangle applies to all images. CTRL: control group; PN: peripheral neuropathy group.

Morphological observations, on specimens collected at the end of treatment, showed a pattern mirroring neurophysiological recordings. Going from proximal to distal, in CTRL animals, the total number of large myelinated fibers physiologically decreases, as the nerves branch out. Instead, in PN animals, a very mild axonal loss was present at the base of the tail and a relevant length-dependent increase in damage severity was confirmed, moving distally. Remarkable axonal damage and an axonal loss, as well as degenerating fibers, were visible starting from the mid of the tail, going from proximal to distal. In the most distal portions of the nerve, axonal degeneration was so severe that only remnants of degenerated fibers were still visible, matching the absence of recordable traces at NCS. Figure 5 shows the most representative sections comparing a CTRL and a PN animal. 

### 2.4. Ultrastructural Examination Microscopy of the Isolated Caudal Nerve

**Figure 6:**
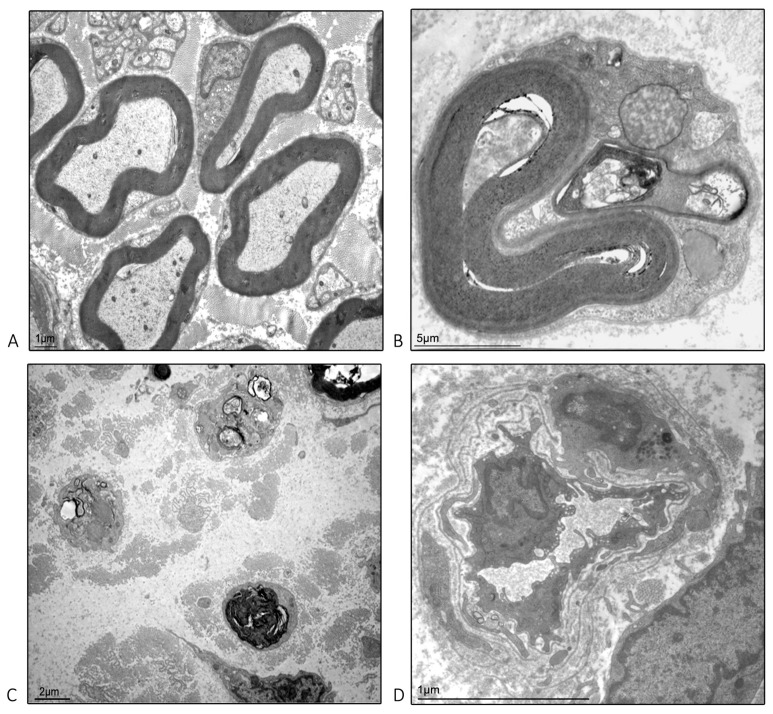
Representative images of length-dependent damage observed in the peripheral neuropathy (PN) group. (**A**): images at the base of the tail. (**B**): images at the mid of the tail. (**C**): images at the tip of the tail. (**D**): a monocyte leaving the vascular compartments to become a tissue macrophage in one of the distal portions.

On the basis of the observations of epoxy-resin embedded caudal nerves, appropriate caudal nerve segments of the CTRL and PN groups were further investigated with the electron microscope. In the most proximal portion of the nerve of PN animals (Figure 6A), the majority of the fibers still showed a normal appearance, whereas at the level of the mid-portion of the nerve (Figure 6B) active axonal degeneration was clearly visible. In the most distal portion of the nerves (Figure 6C) a severe axonal loss was demonstrated in PN animals, as well as some residual signs of active axonopathy. In line with our previous studies on PN models based on chronic PTX administration, we observed cells migrating from the endoneurial vessels into the endoneurium, which are likely to become infiltrating macrophages [11] (Figure 6D).

## 3. Discussion

The rat tail is characterized by the presence of four different nerve trunks (two minor dorsal nerves and two major ventral nerves with a marked overlapping in their innervation zones) which travel the whole length of the tail and are related to both motor and sensory innervation. The motor component of the nerve is in charge of the dorsal muscle (they allow dorsolateral extension of the tail), and of the ventral and ventrolateral muscles (they allow the ventrolateral flexion of the tail) [12]. Caudal nerves are easily accessible and are the ideal site for performing both NCS and peripheral specimen harvesting for morphological observations. 

The light microscopy analyses of the paraffin-embedded whole tails demonstrated that damage observed in PN epoxy-resin embedded caudal nerves was actually due to systemic toxicity, ruling out a local toxic effect of repeated iv injections of a drug that can damage the surrounding tissues if inadvertently extravasated. For the observation of the isolated caudal nerve, we selected the ventral ones since they are larger and, therefore, more suitable to be studied for morphological analysis [13]. Compared to the previous study by Canta et al. [12], here we provide a much more extensive neurophysiological and morphological characterization of the caudal nerves, with serial assessment along the entire length of the tail. We provide the measurement of the SNAP amplitude and not just the SCV. Accordingly, we also expanded on the previous observations of Schaumburg et al. [4] who applied limited neurophysiological testing, also lacking SNAP amplitude calculation, a pitfall in common with some other previously published works [14,15]. This methodological aspect is highly relevant since SNAP amplitude is the crucial parameter to detect axonal damage because it mirrors the number of fibers that constitute the nerve and are functionally active [16].

To provide useful information to devise, in the future, more refined studies investigating axonal damage, we used as a positive reference a well-described animal model of length-dependent axonopathy induced by PTX. In this model, very distal portions of the nerve show severe damage, while very proximal assessment might be unable to detect significant changes [10,17,18,19,20], eventually missing the progressive increase in axonopathy severity. The more detailed characterization provided in this study allowed us to follow the increase in damage severity pairing NCS and morphological observations, and, therefore, this neurophysiological protocol better characterized the severity and length-dependency of axonal damage compared to our previous shorter protocol [10].

## 4. Materials and Methods

### 4.1. Animals and Housing

Sixteen female Wistar rats (175–200 g) were purchased from Envigo Laboratory (Udine, Italy) and housed in a certified animal facility characterized by a tightly monitored light cycle (i.e., environmental light is kept off for 12 h and then off for 12 h), a constantly monitored environmental temperature (i.e., kept at 22 ± 2 °C with 50 ± 20% of relative humidity). Rats were housed with food and water ad libitum. Clinical conditions were monitored daily and body weight was monitored once a week, before each drug treatment.

### 4.2. Drugs

Animals were divided into a CTRL (vehicle-treated) and a PN group (n = 8 animals/group); in the latter, PN was induced via PTX repetitive administrations. Rats were treated once a week for 4 weeks with vehicle or PTX solution, intravenously (iv) in the caudal vein. Solutions were freshly prepared at each administration. The vehicle solution was composed of 80% saline, and 10% between 80 and 10% EtOH absolute. PTX (10 mg/kg, LC laboratories, Boston, MA, USA) was dissolved in the vehicle solution as previously described in detail [21]. 

### 4.3. Study Design

At baseline, NCS were performed to ensure homogeneity between the 2 groups, and analyses were carried out again at treatment completion. Subsequently, animals were sacrificed and specimens for morphological analyses were collected. 

### 4.4. Nerve Conduction Studies (NCS) of the Caudal Nerve for the Whole Length of the Tail

**Figure 7:**
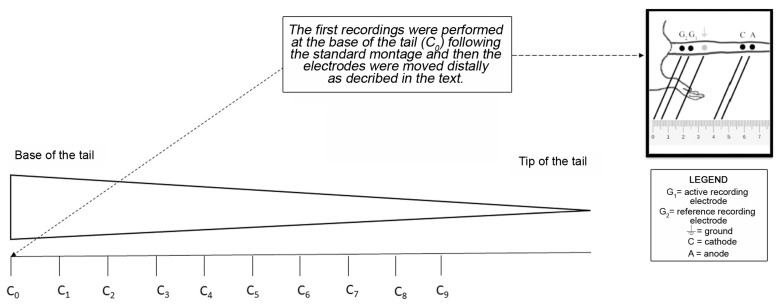
Neurophysiological recordings montage of the caudal nerve for its whole length. The image represents the whole tail of the rat. The C_0_ site (i.e., base of the tails) was the starting point of recordings. In the upper right part of the image the reciprocal position of anode, cathode, active and recording electrodes, and ground electrodes is shown. The measurements were repeated moving the electrodes distally, keeping the interelectrode distances constant.

We refined the recording setup we previously described [10] to extend the neurophysiological testing beyond the proximal and distal portions of the caudal nerve only. Recordings were performed on all animals at baseline and at the end of treatment. Electromyography apparatus Matrix Light (Micromed, Mogliano Veneto, Italy) and stainless-steel needle electrodes were used (Subdermal EEG needle, AmbuTM, Ballerup, Denmark). All procedures were performed under standard conditions and deep isoflurane anesthesia: animal body temperature was monitored and kept constant at 37 ± 0.5 °C with a thermal pad, electronically connected to a thermal rectal probe (Harvard Apparatus, Holliston, MA, USA). Recordings were performed in an orthodromic setting. To record SNAP, the reference recording electrode was first placed at the base of the tail (C_0_) and the active recording electrode 1 cm from it, the cathode was placed 3 cm from the active recording electrode, and the anode was placed 4 cm from it, while the ground electrode was placed midway between the cathode and the active recording electrode. Then, the recordings were serially repeated translating the electrodes distally towards the tip of the tail, keeping constant the interelectrode distances described above (e.g., then placing the most proximal electrode at 1 cm from the base of the tail at C_1_, then at 2 cm from the base of the tail at C_2_, …), as shown in Figure 7. Intensity, duration, and frequency of stimulation were set up to obtain optimal results. The peak-to-peak amplitude was considered. For SCV calculation, SNAP onset was considered. Filters were kept between 20 Hz and 3KHz, and the sweep was kept at 0.5 ms.

### 4.5. Sample Harvesting and Processing for Light and Electron Microscopy

Whole tails were harvested and fixed as a whole piece in 10% formalin solution for 7 days. Specimens were collected from 4 animals per group. After fixation, samples were placed in a 7% ethylenediaminetetraacetic acid (EDTA) solution for 15 days to obtain adequate bone decalcification. At the end of the decalcification process, 1 cm-segments, corresponding to sites of the end of treatment NCS recordings were obtained and then were paraffin embedded: alternate 1 cm apart sections, starting from the proximal end of the nerve (C_0_) were thus prepared, for a total of 8 segments (i.e., C_0_, C_2_, C_4_, …). Transverse sections (4 μm thick) were obtained and stained with hematoxylin and eosin for light microscopy observations.

**Figure 8:**
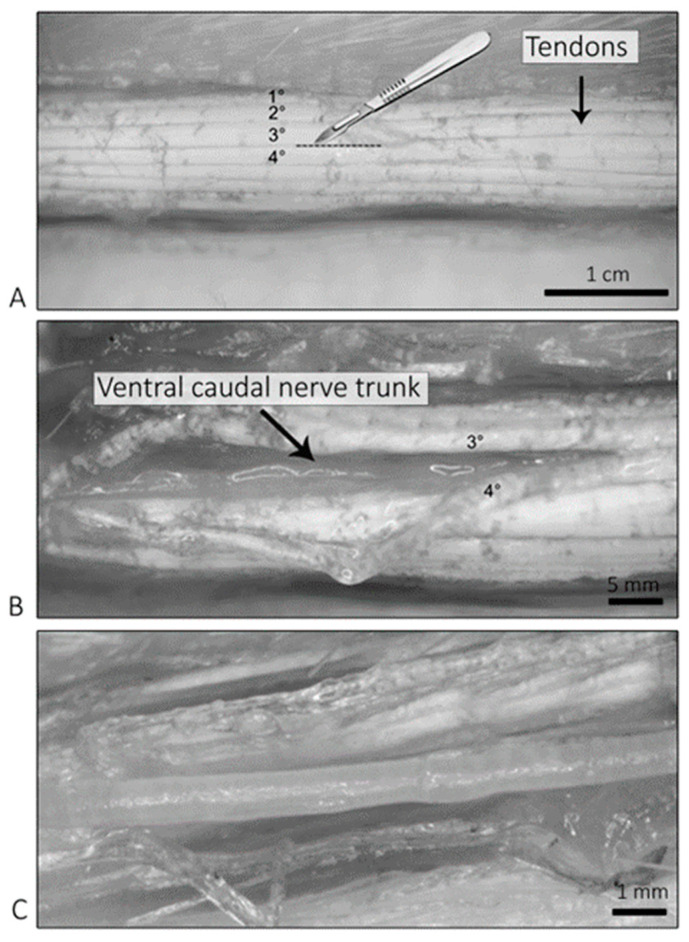
Harvesting procedure. In (**A**) tendon, localization is shown. In (**B**) the exposed ventral caudal nerve, after displacing the third and the fourth tendon, is shown. In (**C**), a close-up of the isolated nerve is shown.

Isolated ventral caudal nerves were harvested from the remaining animals as a single piece. To isolate them, the skin was cut and peeled off starting from the most proximal part of the rat tail and going distally. Using a stereomicroscope (Motic SMZ-178, Seregno, Italy) with 0.8x zoom magnification, the ventral caudal nerves were identified using as a reference the lateral tendons of the tail: the nerve, in fact, is usually found lying below the third and the fourth tendon (numbers are assigned counting from the top), as shown in Figure 8.

Nerves were then carefully pinned on a smooth surface with fine needles both at the distal and proximal ends to keep them straight during the fixation process, carefully avoiding any stretching; fixation was obtained via immersion in 3% glutaraldehyde at room temperature for 3 h. After that, the whole nerves were subdivided into 1 cm segments corresponding to sites of the end of treatment NCS recordings, post-fixed in OsO4 and embedded in epoxy resin; therefore, alternate 1 cm apart sections, starting from the proximal end of the nerve (C_0_), were obtained for a total of 8 segments, similar to what was carried out for the whole tails as described above. 

### 4.6. Light Microscopy Observations—Whole Tails

Morphological analysis was performed on 4 μm thick transverse sections stained with hematoxylin and eosin using a Nexcope NE920 light microscope (TEsseLab, Milano, Italy). Representative images were captured with an ex-Focus-0.66x digital camera (MIchrome, Gaishan Town, Fuzhou, China). All samples obtained from each animal were analyzed.

### 4.7. Light Microscopy Observations—Isolated Caudal Nerves

Morphological analyses were performed on 1.5 µm thick semithin sections stained with toluidine blue using a Nexcope NE920 light microscope. Images were obtained with the equipped ex Focus-0.66× digital camera (MIchrome, Gaishan Town, China). At least 2 tissue blocks per segment were analyzed per each animal.

### 4.8. Electron Microscopy Observations—Isolated Caudal Nerve

Based on the light microscopic findings, unstained ultrathin sections were prepared from selected tissue blocks and examined with a LVEM 25 (De Long, Brno, Czech Republic) transmission electron microscope.

### 4.9. Statistical Analysis

Sample size was calculated on the basis of SCV reference values, as previously reported [10,22]. Briefly, the relevant difference against CTRL group was set at 5 m/s and we considered the standard deviation for SCV as = 3: with a 2-sided 5% alpha and an 80% power, the sample size is 7 animals/group (www.dssresearch.com/KnowledgeCenter/toolkitcalculators/samplesizecalculators.aspx, accessed on 7 September 2021). We slightly increased (n = 8), the sample size to take into account possible loss of animals due to chemotherapy administration. Descriptive statistics were generated for all variables. Normally distributed data were analyzed with parametric tests (*t*-test) and non-normally distributed with non-parametric tests (Mann-Whitney U-test). Two-sided tests were used. A *p*-value < 0.05 was set as significant. All analyses were conducted in GraphPad environment (v4.0).

## 5. Conclusions

In conclusion, our extensive morphological characterization confirms that the neurophysiological investigation of the caudal nerve is a reliable technique to evaluate animal models of axonopathy, but also that a flexible experimental paradigm should be considered, particularly if the expected (or the observed) severity is relevant. Therefore, in future studies, the mid-tail could be suggested as the ideal site to detect axonal damage, where severity can be remarkable, but not as extreme as at the tip or too mild as at the base of the tail. 

## Figures and Tables

**Table 1:** Distribution of NCS values of the whole population at baseline.

**Caudal Nerve SNAP Amplitude**										
**Recording Site**	**C_0_**	**C_1_**	**C_2_**	**C_3_**	**C_4_**	**C_5_**	**C_6_**	**C_7_**	**C_8_**	**C_9_**
Median (Q1, Q3)	122.0 (109.6, 138.7)	105.6 (91.67, 128.7)	94.21 (86.71, 105.6)	88.83 (74.04, 102.1)	77.56 (72.07, 92.84)	75.85 (68.86, 89.44)	68.94 (58.13, 79.88)	62.79 (56.90, 72.25)	59.02 (50.23, 63.86)	57.14 (52.31, 61.97)
**Caudal Nerve Sensory Conduction Velocity**										
**Recording Site**	**C_0_**	**C_1_**	**C_2_**	**C_3_**	**C_4_**	**C_5_**	**C_6_**	**C_7_**	**C_8_**	**C_9_**
Median (Q1, Q3)	41.86 (40.98, 44.71)	42.74 (40.98, 44.71)	40.15 (39.32, 43.72)	39.32 (37.83, 40.98)	37.83 (37.83, 40.98)	37.83 (36.41, 40.15)	37.83 (35.77, 37.83)	36.41 (34.51, 37.12)	35.77 (33.90, 36.41)	30.71 (29.79, 32.75)

SNAP: sensory nerve action potential; Q1: 25% percentile; Q3: 75% percentile.

## Data Availability

Data will be made available upon request to the corresponding author.
